# Editorial: Biology of brain disorders—Cellular substrates for disrupted synaptic function and experience-dependent plasticity

**DOI:** 10.3389/fncel.2022.947926

**Published:** 2022-08-10

**Authors:** Daniela Tropea, Annalisa Scimemi, Abhishek Banerjee

**Affiliations:** ^1^Department of Psychiatry, Trinity College Dublin, Dublin, Ireland; ^2^FutureNeuro Research Centre, Science Foundation Ireland, Dublin, Ireland; ^3^Trinity College Institute for Neuroscience, Trinity College Dublin, Dublin, Ireland; ^4^Trinity Translational Medicine Institute, Trinity College Dublin, Dublin, Ireland; ^5^Department of Biology, University at Albany, Albany, NY, United States; ^6^Biosciences Institute, Newcastle University, Newcastle upon Tyne, United Kingdom

**Keywords:** synaptic plasticity, neurodevelopmental disorders, neurodegenerative disease, mouse models, brain function, molecular pathways

This Research Topic on the “*Biology of Brain Disorders: Cellular Substrates for Disrupted Synaptic Function and Experience-Dependent Plasticity*” is a continuation of a series of topics and conferences on Brain Disorders that started in 2016. The topic aims to highlight the convergences and divergences between different types of brain disorders, including neuropsychiatric, neurological, and neurodegenerative. These pathologies remain one of the major problems in healthcare, and their incidence has continued to grow over the years. Consequently, one of the challenges that have captivated neuroscientists for decades is developing and exploiting sophisticated experimental approaches to understand how brain disorders arise and affect different features of brain function, including changes in synaptic transmission and plasticity. Indeed the field of neuroscience, among other areas of health sciences, has witnessed great technological advancements in the last several years, and has emerged as a positive circle of discovery and understanding: as new technologies are being developed, new mechanisms are discovered, and the need to understand the operating principle of such mechanisms in specific neuronal connections stimulates more research.

Aside from these technological advances, the use of animal models remains a classic approach to studying brain disorders. In this Research Topic, several disease models rely on genetically-engineered mice and models of brain injury. Pak et al. analyze how visual perception changes in a mouse model of the Fragile-X syndrome. Using a more classical approach, Cui et al. show how changes in Ca^2+^ channel subunit composition alter the synaptic plasticity of nociceptive pathways. In another study, Xu et al. present data collected from a rat model of chronic intermittent hypoxia. In brain injury, *in vitro* Ca^2+^ imaging reveals that the high-frequency head impact protocol (HFHIP) alters synaptic plasticity, resulting in a decreased coordination in the activity of neuronal ensembles (Chapman et al.). Further evidence of altered neuroplasticity due to brain injury is provided by Xu et al. and McDaid et al., using *in vivo* and *in vitro* studies.

While animal models have been extremely useful in advancing our understanding of brain function in health and disease, the translational potential of mouse models remains a general concern for applications in human patients. We acknowledge that this issue arises from the multiple failures of clinical trials at the advanced stage, despite the positive outcome of preclinical studies. Scientists are therefore discussing the validity of animals in studying the mechanisms of human disorders and recognizing the necessity to identify and focus on the specific endophenotypes that are present both in the animals and in patients. Often these endophenotypes are related to synaptic function and plasticity, as is the case for Huntington's disease and schizophrenia, which are linked to altered synaptic transmission in human and animal models (Barron et al.; Navarrete and Zhou). While the aforementioned works identify deficits in synaptic function that affect all cells, other disorders lead to patterned cell death only in specific cell types, as happens for Purkinje cells in patients and animal models of autosomal recessive spastic ataxia of Charlevoix-Saguenay (Toscano Márquez et al.).

To fill the gap between preclinical and clinical studies, and avoid differences among species, additional strategies have been developed for translational and clinical research. Cellular models consisting of patient-derived cells and organoids have been generated, and these preparations hold the promise of advancing our understanding of the molecular mechanisms disrupted in diseases, linking the clinical presentation to deficits in neurodevelopmental processes (de Paula Moreira et al.). This approach had several limitations initially, spanning from a lack of insights at the systems level to the limited molecular and cellular endophenotypes that can be measured. The field is growing fast, and these cellular models are now becoming an indispensable tool for translational research. In our view, animal models and patient-derived cellular preparations are complementary models for understanding the pathophysiology of brain disorders.

A theme that has emerged in recent years is the identification of convergences across disorders. Numerous genetic studies during the whole genomic sequencing era provided important contributions to understanding of the molecular basis of brain disorders, but they also showed that multiple common genes are associated with several pathologies. It is now clear that genetic studies alone are insufficient to identify molecular targets of intervention for specific brain disorders. The convergence of pathways across disorders is reported in this topic at a molecular and functional level. For example, RNA6 is involved in both epilepsy (Mathoux et al.) and Alzheimer's disease (Zhang et al.), while the review by Bach et al. examines the common mechanisms present at the circuitry level between Fragile-X and Rett syndrome. The overlap of genes and mechanisms suggests the possibility of ameliorating different disorders using similar therapeutic intervention strategies.

In the era of data science, we cannot ignore the contribution of big data in revealing the complexities of brain disorders. Indeed, in the paper by Chatterjee et al., computational analysis is used to process all existing functional data to generate predictions of alterations in autism spectrum disorders.

The articles and reviews featured in this Frontiers Research Topic confirm that the study of the genetic and synaptic mechanisms underlying brain disorders can shed light on the mechanisms of brain function. It provides insights on many of the molecular and cellular alterations that ultimately converge to modify the function and plasticity of single synapses and complex neuronal circuits.

## Author contributions

All authors listed have made a substantial, direct, and intellectual contribution to the work and approved it for publication.

## Funding

DT is an investigator in the FutureNeuro Research Centre supported by Science Foundation Ireland (SFI) under Grant Number 16/RC/3948 and co-funded under the European Regional Development Fund and by FutureNeuro industry partners. DT is also supported by Lejeune Foundation (1935–2020), International Rett Syndrome Foundation (IRSF- grant 3507-2017, Meath Foundation (2019 research grant -coPI). AS is supported by grant NSF 2011998, and AB is supported by a Wellcome Trust institutional strategic award and a Royal Society Research Grant.

## Conflict of interest

The authors declare that the research was conducted in the absence of any commercial or financial relationships that could be construed as a potential conflict of interest.

## Publisher's note

All claims expressed in this article are solely those of the authors and do not necessarily represent those of their affiliated organizations, or those of the publisher, the editors and the reviewers. Any product that may be evaluated in this article, or claim that may be made by its manufacturer, is not guaranteed or endorsed by the publisher.

